# Dawning public health services dogma: An indigenous Southwest Chinese perspective in managing hypertension-with or without the “BPHS”?

**DOI:** 10.3389/fpubh.2022.1017795

**Published:** 2022-11-10

**Authors:** Linhong Pang, Lakshme Kottu, Zihong Guo, Yi Shi, Misbahul Ferdous, Yajing Zhao, Mingjing Tang, Wei Liu, Jiayu Fang, Hongchen Fu, Xia Wu, Min Ma, Huadan Wang, Daphne Merkus, Lin Duo

**Affiliations:** ^1^Affiliated Cardiovascular Hospital of Kunming Medical University, Fuwai Yunnan Cardiovascular Hospital, Kunming, China; ^2^School of Public Health, Kunming Medical University, Kunming, China; ^3^Division of Experimental Cardiology, Erasmus University Medical Center, Rotterdam, Netherlands; ^4^National Clinical Research Center for Cardiovascular Diseases, State Key Laboratory of Cardiovascular Disease, Fuwai Hospital, National Center for Cardiovascular Diseases, Chinese Academy of Medical Sciences and Peking Union Medical College, Beijing, China; ^5^Walter Brendel Center of Experimental Medicine (WBex), LMU Munich, Munich, Germany

**Keywords:** basic public health service, hypertension management, effectiveness and quality, low and middle-income countries, health policy

## Abstract

**Background:**

To alleviate the rising mortality burden due to hypertension and other non-communicable diseases, a new public health policy initiative in 2009 called the Basic Public Health Services (BPHS). Program was introduced by the Chinese government. The goal of the study is to assess the feasibility and impact of a nationwide health care service—the “BPHS”.

**Methods:**

From January to December 2021, a stratified multistage random sampling method in the survey was conducted to select 6,456 people from 8 cities/districts in Yunnan Province, China, who were above the age of 35 years. 1,521 hypertensive patients were previously aware of their high blood pressure status were matched to the BPHS program database based on ID number and then further divided into BPHS group and non-BPHS (control) group. The results of the current study are based on their responses to a short structured questionnaire, a physical examination, and laboratory tests. The association between BPHS management and its effect on the control of hypertension was estimated using multivariable logistic regression models. We evaluated the accessibility and efficacy of BPHS health care services by analyzing various variables such as blood pressure, BMI, lifestyle modification, anti-hypertensive drugs taken, and cardiovascular risk factors.

**Results:**

Among the 1,521 hypertensive patients included in this study, 1,011 (66.5%) were managed by BPHS programme. The multivariable logistic regression model demonstrated that the BPHS facilitated hypertension control (OR = 1.640, 95% CI: 1.237–2.175). A higher proportion of participants receiving lifestyle guidance from the BPHS management showed lowering of total cholesterol. In comparison to the non-BPHS group, those under BPHS management adhered better to antihypertensive medications either single drug (54.3%) or in combination (17.3%) of drugs. Additionally, we also noticed that urban areas with centralized and well-established digital information management system had better hypertension treatment and control.

**Conclusions:**

Nearly two-thirds of the hypertensive patients in Yunnan Province were included in BPHS management. The impact of the national BPHS program was evident in lowering risk factors for cardiovascular diseases, promoting healthy lifestyles, lowering blood pressure, increasing medication adherence, and the better control rate of hypertension.

## Introduction

The number of people with hypertension increases from 650 to 1.28 billion worldwide from 1990 to 2019 in the age group of 30–79 years (1). Hypertension has become the major cause of premature mortality and cardiovascular disease (CVD) globally (2). The Global Burden of Disease Study reported that hypertension was the primary risk factor for all deaths globally, accounting for 19.2% (10.8 million) of all mortality worldwide in 2019 (3). About 245.5 million people are affected by hypertension in China (4), and hypertension is estimated to cause 2.54 million deaths in 2017, with 95.7% of these deaths being due to CVD (5).

Controlling blood pressure to a normal state reduces the risk of cardiovascular events and all-cause mortality (6–8). The trend of cardiovascular disease will be directly impacted by how hypertension is managed in primary medical and health organizations (community health centers, township health centers, and village clinics) (9). Global studies have demonstrated that primary health care (PHC) was better effective in hypertension management and improved blood pressure control (10–14).

In 2009, China's new healthcare reform introduced the “National Basic Public Health Service Program” (BPHS), which provides free of cost health services throughout the country by partnering with community health organizations. The management of hypertensive patients aged 35 and over was one of the 12 kinds of voluntary free services contents in BPHS (15, 16), including screenings, lifestyle guidance for hypertensive patients, at least four in-person follow-up visits per year, risk factor intervention, health education promotion, health examination, referral services, guidance on the use of antihypertensive medicine, and personal health record establishment (17, 18). With BPHS, 35.1% of hypertensive patients have received four or more follow-up assessments in the past 1 year (19), uncontrolled hypertension was reduced by 26% (20). However, most of the earlier studies on being covered by BPHS were self-reported by participants, and focused on the rate of service delivery from primary doctors (21, 22), patient service satisfaction (23, 24) and the community health management rate of hypertensive patients (25, 26). Previous studies also showed a low prevalence of combination therapy and limited compliance with hypertension drug treatment (27, 28). Additionally, the hypertensive control rate is only 15.3% in China (4), and more than 50% of hypertension patients had multiple CVD risk factors, which can affect hypertension control (27, 29).

This study comprehensively evaluated the BPHS hypertension management in Yunnan Province, a relatively economically backward in southwest China. Hypertensive patients who were aware of their high blood pressure from a representative sampling survey and BPHS system matched, to compare matched BPHS group and unmatched non-BPHS groups' coverage and current hypertension status, CVD risk factors, management and effectiveness, blood pressure control rate and antihypertensive drug use, etc. This study supports the World Health Organization's (WHO) suggestion that developing nations should increase their access to management-controlled hypertension-related healthcare services (30). Rarely are there extensive comparisons of BPHS and non-BPHS groups' effects on hypertension management based on unique ID matching published.

## Methods

### Study design and sampling procedures

**Figure 1:**
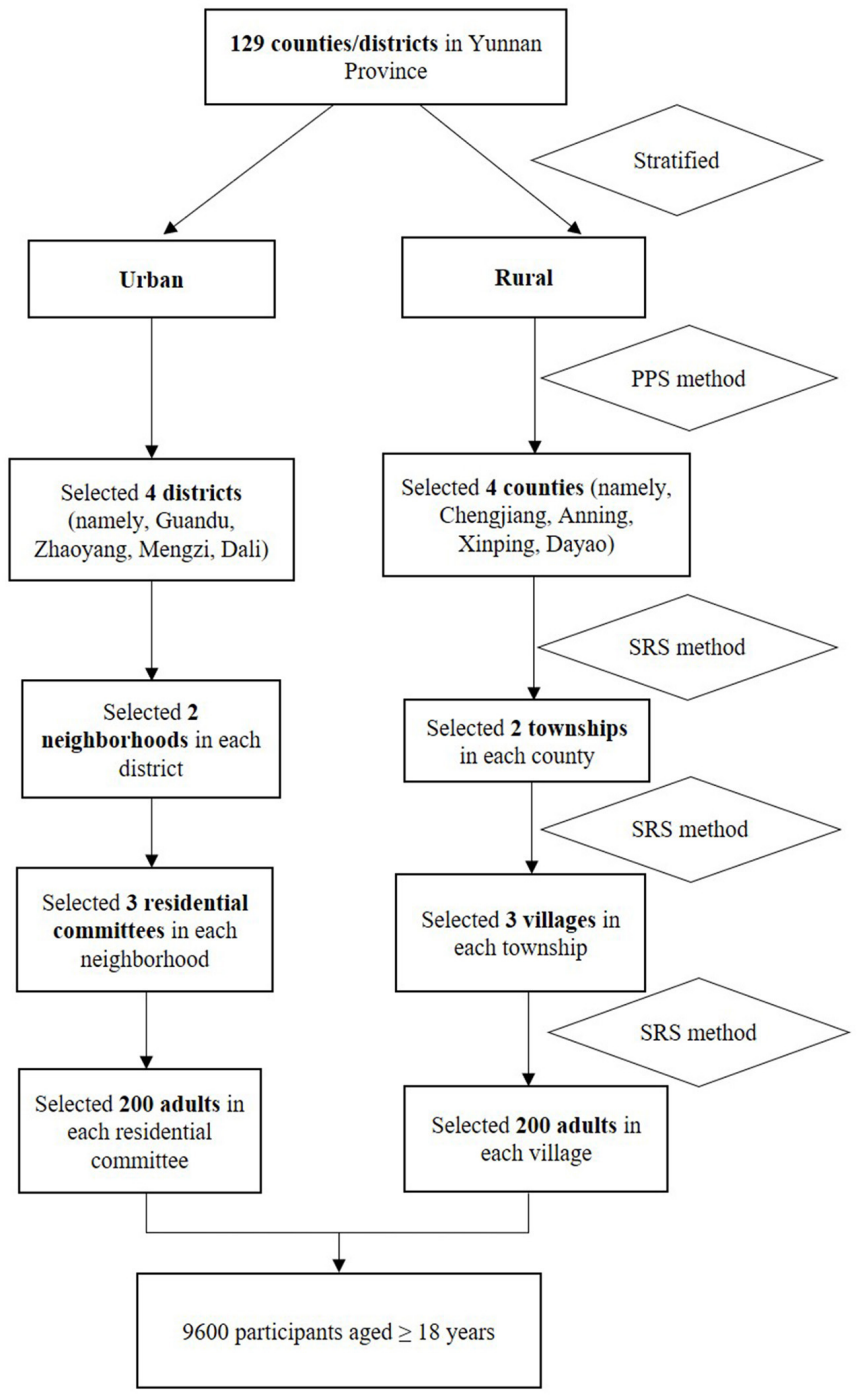
The Hypertension survey procedure of sampling. PPS, probability proportional to size; SRS, simple random sampling.

The survey was conducted in Yunnan province from January to December 2021, and samples were chosen using a stratified multistage random sampling method. As shown in Figure 1, all 129 counties and districts were separated into urban and rural sectors in the initial phase. The probability proportional to size (PPS) sampling was used to choose four districts in the urban areas (namely, Guandu, Zhaoyang, Mengzi, and Dali) and four counties in the rural areas (namely, Chengjiang, Anning, Xinping, and Dayao). Then, two neighborhoods or two townships were randomly sampled in each district or county, respectively. Later, three residential committees or villages were randomly selected within each neighborhood and township, respectively. Finally, 9,600 individuals aged ≥18 years were selected from each chosen residential committee or village by the SRS method after considering the sex and age composition.

**Figure 2:**
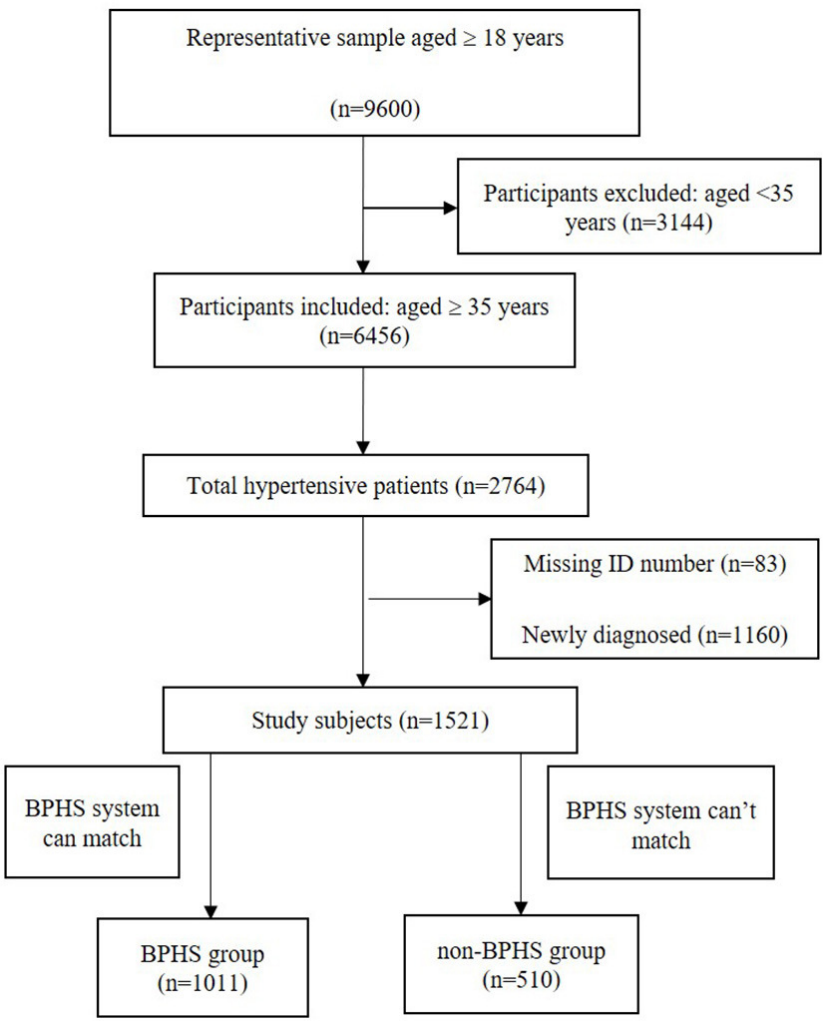
Flowchart of the participant selection process. BPHS, basic public health services.

The target population for BPHS hypertension management is patients who are residents of the jurisdiction aged 35 years and older (17), so we excluded those who did not meet this age. Yunnan Province has established the BPHS electronic case system and hypertension case management package, and patients' follow-up information has been uploaded to the database by primary care physicians based on the patient's unique ID card. Among the population aged ≥35 years 1,521 hypertensive patients who know their high blood pressure status, the unique ID number was matched with the BPHS electronic system, of which 1,011 patients were matched and included in BPHS hypertension management and 510 patients were not included, who were allocated to non-BPHS group (Figure 2).

### Data collection

After receiving sufficient training, the medical staff will serve as the investigation team, using a unified work plan and investigation equipment. After informed consent, basic information was gathered *via* face-to-face short structured questionnaires that included details of their socio-demographics like age, gender, ethnicity, place of residence, educational attainment, occupation, annual household income, and wellness behaviors like smoking, drinking, dieting, exercising, and finally about hypertension perse like the history of hypertension, treatment followed from the time of diagnosis. Additionally, we measured height with RGZ-160 measuring instrument (Jiangsu Suhong Medical Instruments Co., Ltd., Jiangsu, China), and body weight was measured with an InBody H20B (InBody Co., Ltd., Seoul, South Korea) removing shoes, hats, coats, or weight in pockets. Body mass index (BMI) is a person's weight (kg) divided by the square of height (m) (31).

Diastolic blood pressure (DBP) and systolic blood pressure (SBP) were measured in the right upper arm using an OMRON HBP-1,300 (Omron Healthcare Co., Ltd., Kyoto, Japan) at 5 min intervals; three readings were recorded and the average of the three measurements was used for this study. In the meantime, participants were asked to fast for more than 8 h before collecting 8 ml of blood samples for cryopreservation and then sent to Beijing ZhongtongLanbo Medical Test Laboratory for measurement of triglycerides (TG), high-density lipoprotein cholesterol (HDL-C), low-density lipoprotein cholesterol (LDL-C), and total cholesterol (TC). The findings and readings were recorded on the iPad in specially designed survey tables (see Supplementary File 1), and the investigation process was summarized in Supplementary File 2 and Supplementary Video.

### Definition

According to the 2018 Chinese Guidelines (29) and 2020 International Society of Hypertension (ISH). Global Hypertension Practice Guidelines (27), hypertension was defined as mean SBP ≥140 mm Hg and/or mean DBP ≥90 mm Hg, or self-reported use of blood pressure control drugs in the past 2 weeks. Hypertension control was defined as mean SBP <140 mm Hg and DBP <90 mm Hg (29). The antihypertensive drug classification was also consistent with the guidelines. Based on the Chinese guidelines on prevention and treatment of dyslipidemia in adults (32), defined high TG, high TC, high LDL-C, and low HDL-C as ≥2.26, ≥6.22, ≥4.14, < 1.04 mmol/L, respectively.

People who smoked more than one cigarette per day for more than 6 months were considered “current smokers” (33). Participants drinking alcohol at least once a week were classified as “current drinkers” (34). <150 min of moderate-intensity physical activity per week was defined as insufficient physical activity (35). Participants who consumed an average of <400 g of fruits and vegetables per day were insufficient intake (36). According to the recommended criteria for obesity in Chinese (31), overweight was defined as a person's BMI between 23 and 24.9 kg/m^2^, obesity was a BMI of 25 kg/m^2^ and above. Risk factors for cardiovascular disease (CVD) as per 2020 ISH Guidelines (27) were people who smoke, drink, don't eat enough vegetables and fruits, don't get enough exercise, are overweight, or are obese.

### Statistical analyses

All statistical analyses use IBM SPSS 22.0 (SPSS Inc., New York, NY, USA). Categorical variables from the BPHS and non-BPHS groups were presented as numbers (proportions), and differences between groups were compared using the chi-square test or Fisher's exact test. Mean ± standard deviation (SD) and *t*-tests were used for normally distributed continuous variables. Four indicators of blood lipids (TG, TC, HDL-C, and LDL-C) were skewed distribution data by Kolmogorov-Smirnov test, described by median and inter quartile range, and the differences between groups were tested by Wilcoxon rank sum test.

Eleven characteristic variables as age (35–44, 45–54, 55–64, and ≥65 years), residence (urban and rural), gender (male and female), ethnicity (Han or other minorities), annual household income (<4,487 USD/year, ≥4,487 USD/year), education attainment (elementary school and below, junior high school and above), current smoker (yes or no), current drinker (yes or no), insufficient vegetable and fruit intake (yes or no), insufficient physical activity (yes or no), overweight and obesity (yes or no) were compared between the two groups.

The ggplot2 package of R 4.0.5 (The R Project for Statistical Computing, Vienna, Austria) was used to construct the visible distribution of blood pressure and a stacked column chart of “controlled with treatment”, “treated but not controlled”, and “diagnosed but untreated”. Unadjusted and full adjusted binary logistic regression models were conducted to assess the association between hypertension control with BPHS, the first model did not adjust for potential influencing factors. Next, second model was performed to adjust for potential influencing factors, including age, gender, ethnicity, residence, household income and educational level. Then, the odds ratio (OR) and 95% confidence interval (CI) were estimated. All tests were two-sided and *p* < 0.05 was considered statistically significant.

## Results

### General description of participants

The mean age of the 1,521 hypertensive patients was 62.6 ± 12.6 years old, with 737 men (48.5%) and 858 people living in urban areas (56.4%), 1,118 Han people (73.5%), 1,104 people (72.6%) with an annual family income of <4,487 USD/year, and 898 people (59.0%) with primary school education or below (Table 1).

**Table 1:** Socio-demographic characteristics and distribution of CVD risk factors between the BPHS and non-BPHS groups in Yunnan, China.

**Characteristics**	**Total (*n* = 1,521)**	**BPHS group (*n* = 1,011)**	**non-BPHS group (*n* = 510)**	***p*-Value**
**Demographic and socioeconomic characteristics**			
Age, (mean ± SD)^£^	62.6 ± 12.6	65.0 ± 12.1	57.8 ± 12.3	
35–44	134 (8.8)	52 (5.1)	82 (16.1)	<0.001
45–54	324 (21.3)	179 (17.7)	145 (28.4)	
55–64	365 (24.0)	233 (23.0)	132 (25.9)	
≥65	698 (45.9)	547 (54.1)	151 (29.6)	
Male sex	737 (48.5)	455 (45.0)	282 (55.3)	<0.001
Urban residence	858 (56.4)	558 (55.2)	300 (58.8)	0.178
Han people	1,118 (73.5)	716 (70.8)	402 (78.8)	0.001
Household income < 4,487 USD/year	1,104 (72.6)	771 (76.3)	333 (65.3)	<0.001
Education, elementary school and below	898 (59.0)	654 (64.7)	244 (47.8)	<0.001
**CVD risk factors**				
Current smokers	223 (14.7)	135 (13.4)	88 (17.3)	0.042
Current drinkers	267 (17.6)	177 (17.5)	90 (17.6)	0.946
Insufficient vegetable and fruit intake	1,330 (87.4)	869 (86.0)	461 (90.4)	0.014
Inadequate physical activity	411 (27.0)	282 (27.9)	129 (25.3)	0.281
Overweight and obesity^#^	909 (66.3)	612 (66.0)	297 (66.7)	0.791
**Blood pressure** ^£^				
SBP	147.8 ± 19.0	146.8 ± 18.8	149.7 ± 19.1	0.006
DBP	87.6 ± 12.8	85.7 ± 12.5	91.2 ± 12.7	<0.001
**Blood pressure status**				<0.001
Diagnosed but untreated	563 (37.0)	287 (28.4)	276 (54.1)	
Treated but uncontrolled	510 (33.5)	388 (38.4)	122 (23.9)	
Controlled	448 (29.5)	336 (33.2)	112 (22.0)	

CVD, cardiovascular diseases; SD, standard deviations; BPHS, basic public health services; BMI, body mass index.

# Height or weight data were not available for 226 participants.

£ Mean ± standard deviation, *t*-test was used to compare the mean DBP and SBP of the BPHS group and the non-BPHS group.

### Demographic and CVD risk factors differences between the BPHS and non-BPHS groups

1011 (66.5%) and 510 (33.5%) hypertensive patients came from the BPHS and non-BPHS groups. Hypertensive patients included in BPHS management were more likely to be older, female, non-Han Chinese, and had lower family economics and literacy (all *p* < 0.05). Furthermore, the BPHS group had a lower proportion of current smokers and their consumption of fruits and vegetables was inadequate (all *p* < 0.05).

### Blood pressure

**Figure 3:**
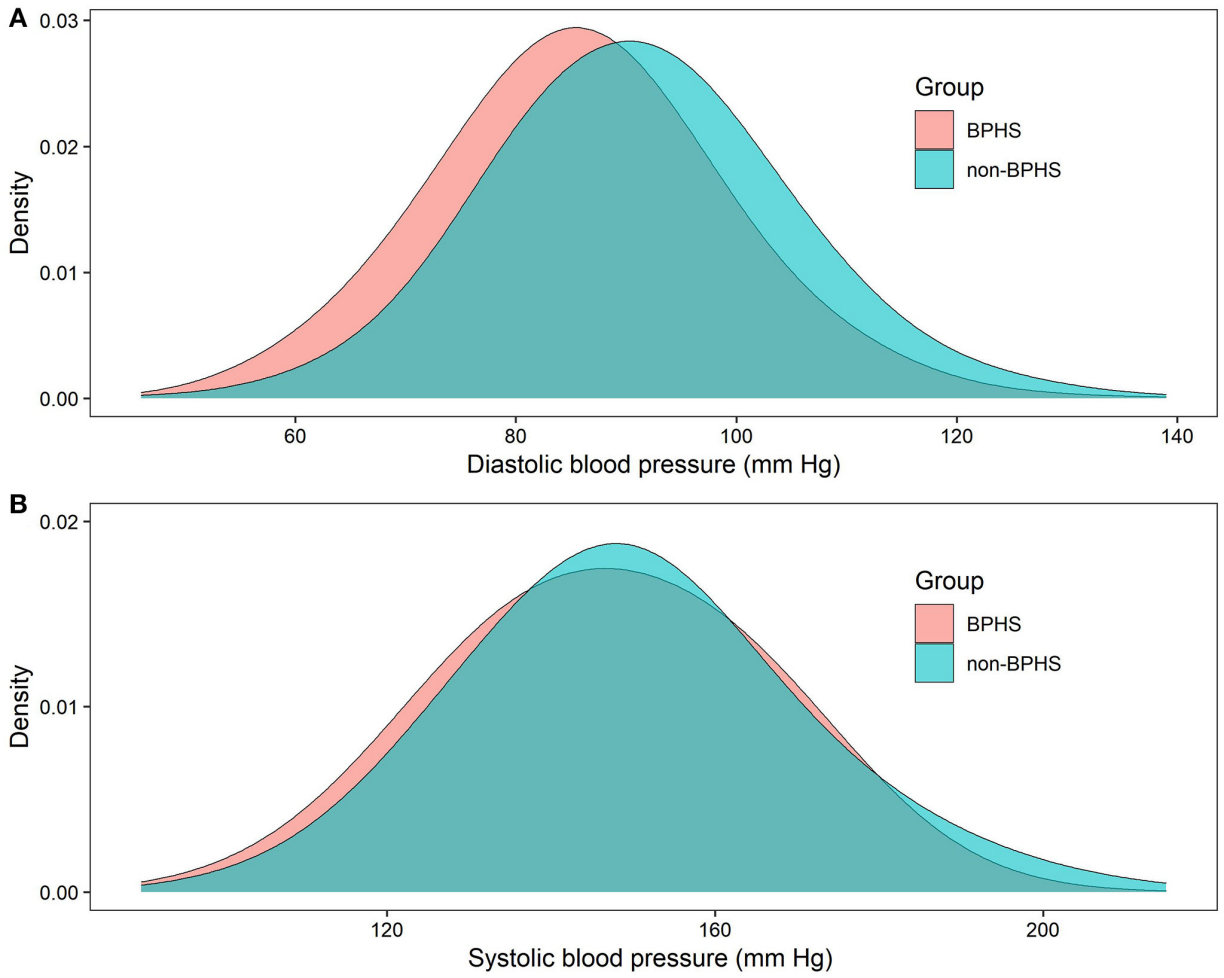
Distribution of DBP **(A)**, SBP **(B)** by BPHS and non-BPHS groups, in Yunnan China.

As shown in Table 1, those who received BPHS management had significantly lower SBP and DBP than those who did not (*p* < 0.001), the average SBP was (146.8±18.8 vs. 149.7 ± 19.1) mm Hg, and DBP (85.7 ± 12.5 vs. 91.2 ± 12.7) mm Hg, respectively (all *p* < 0.05). The non-BPHS group showed greater variation in SBP and DBP distribution than the BPHS group (Figure 3). Furthermore, more than half of the patients did not receive antihypertensive medication in the non-BPHS group.

### Control of hypertension

**Figure 4:**
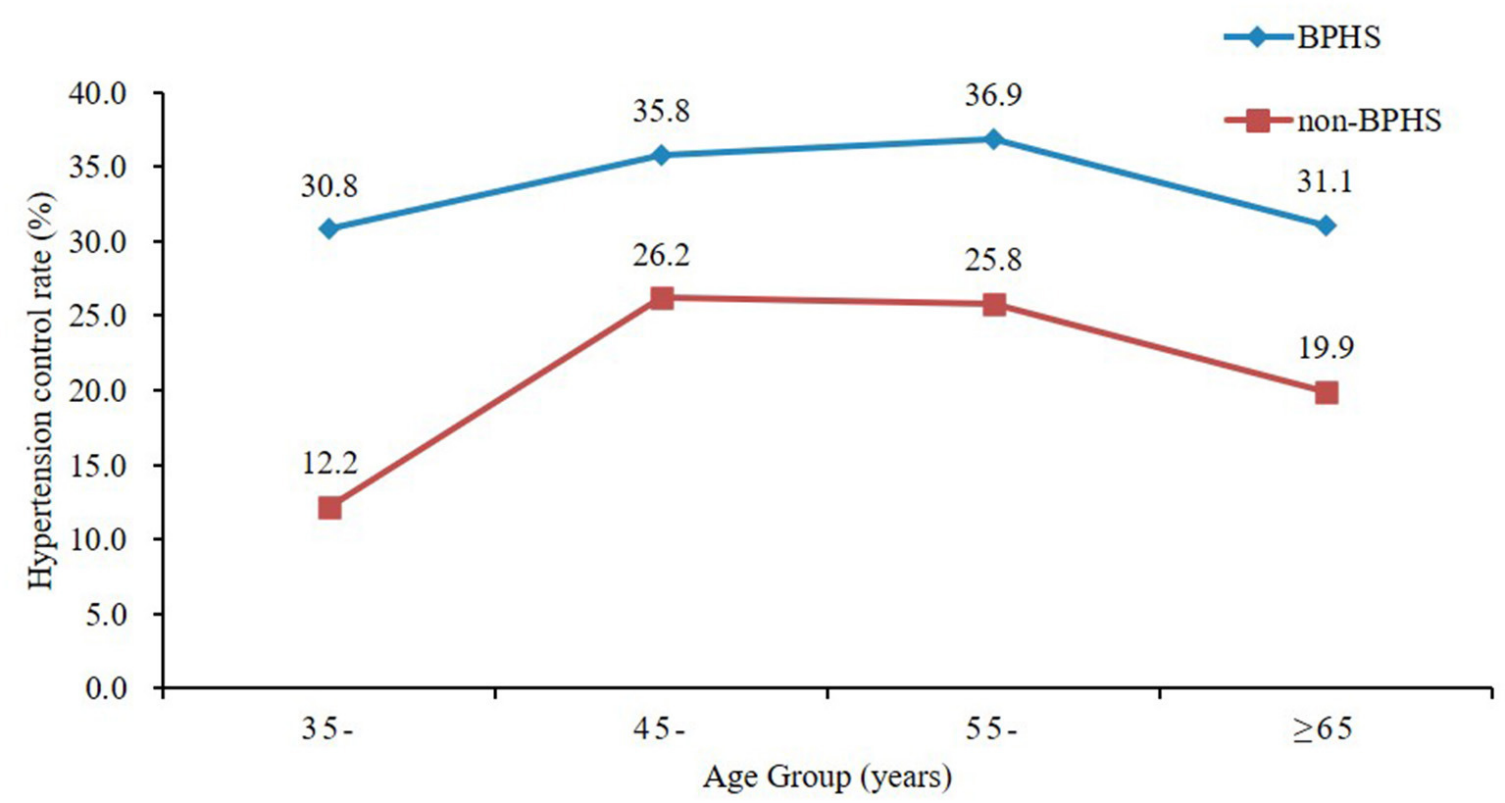
Hypertension control rate by age between the BPHS and non-BPHS groups.

The BPHS group indicated higher hypertension control rate (33.2%) than the non-BPHS group (22.2%) shown in Table 2. Furthermore, model 1 (did not adjusted for independent variables), and model 2 (adjusted confounding variables) all showed a significantly higher hypertension control rate from the BPHS group (OR = 1.640, 95% CI: 1.237–2.175). Regardless of the age, those who received BPHS management had higher rates of hypertension control, as shown in Figure 4.

**Table 2:** Unadjusted and adjusted models: association of BPHS with control of hypertension.

	**Control, *n* (%)**		**Model 1**		**Model 2**		
		***OR***	**95%*CI***	***p*-Value**	***OR***	**95%*CI***	***p*-Value**
non-BPHS group	112 (22.2)	Ref	–	–	Ref	–	–
BPHS group	336 (33.2)	1.769	1.382–2.265	<0.001	1.640	1.237–2.175	0.001

BPHS, basic public health services.

Model 1 did not adjust for potential independent variables.

Model 2 adjusted confounding variables, including age, ethnicity, residence, gender, annual household income, and educational level.

### Comparison of BPHS and non-BPHS group lifestyle modification services and lipid levels

**Figure 5:**
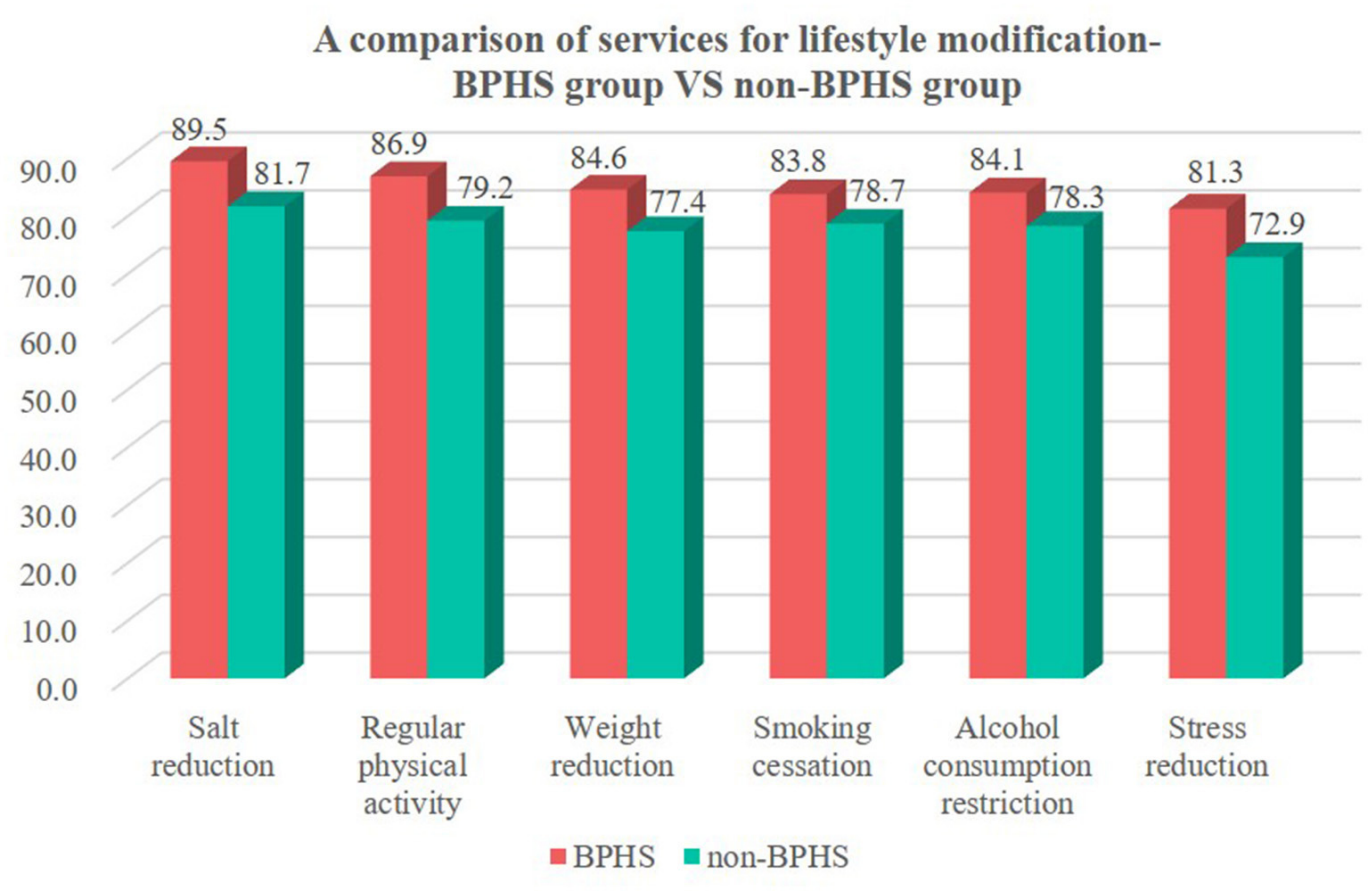
Hypertension patients received lifestyle modification services among BPHS and non-BPHS groups in Yunnan China.

Among those hypertensive patients enrolled in BPHS, the percentages who had received lifestyle modification services on salt reduction guidance, regular physical activity, weight reduction, smoking cessation, alcohol restriction, and stress reduction guidance services were 89.5, 86.9, 84.6, 83.8, 84.1, and 81.3%, respectively, both higher than the non-BPHS group, with 81.7, 79.2, 77.4, 78.7, 78.3, 72.9%, respectively (chi-square test, *p* < 0.05) (Figure 5).

Further analysis proved that patients with BPHS had lower TC after receiving weight reduction guidance, smoking cessation guidance, and alcohol consumption restriction guidance than those who did not receive such services. In addition, patients who received weight loss, smoking cessation, alcohol consumption restriction, and stress guidance also had lower HDL-C, however within the normal range. No significant differences were observed in TG and LDL-C levels between those who received lifestyle modification services and those who did not (Table 3).

**Table 3:** Differences in blood lipid measurements among patients included in the BPHS group.

**Lifestyle modifications**	**TG**	**TC**	**LDL-C**	**HDL-C**
**Salt reduction**				
YES	1.38 (0.93–2.24)	4.97 (4.26–5.65)	3.00 (2.44–3.60)	1.36 (1.17–1.58)
NO	1.50 (1.05–2.20)	5.20 (4.43–5.74)	3.10 (2.52–3.67)	1.43 (1.22–1.63)
*p*-Value	0.277	0.072	0.254	0.059
**Regular physical activity**			
YES	1.50 (1.03–2.19)	4.98 (4.28–5.66)	3.00 (2.45–3.60)	1.36 (1.17–1.58)
NO	1.47 (1.02–2.36)	5.18 (4.31–5.65)	3.05 (2.42–3.64)	1.41 (1.20–1.63)
*p-*Value	0.974	0.290	0.629	0.129
**Weight reduction**				
YES	1.49 (1.03–2.20)	4.93 (4.25–5.63)	2.98 (2.43–3.56)	1.36 (1.16–1.57)
NO	1.50 (1.04–2.36)	5.18 (4.50–5.77)	3.13 (2.52–3.67)	1.43 (1.23–1.65)
*p-*Value	0.832	0.011	0.079	0.009
**Smoking cessation**				
YES	1.49 (1.02–2.16)	4.93 (4.23–5.62)	2.99 (2.42–3.56)	1.36 (1.16–1.57)
NO	1.52 (1.05–2.59)	5.19 (4.47–5.89)	3.05 (2.51–3.66)	1.43 (1.21–1.62)
*p-*Value	0.251	0.008	0.121	0.022
**Alcohol consumption restriction**			
YES	1.49 (1.02–2.17)	4.93 (4.25–5.63)	3.00 (2.42–−3.58)	1.35 (1.16–1.57)
NO	1.52 (1.04–2.57)	5.21 (4.51–5.76)	3.11 (2.54–3.67)	1.43 (1.22–1.62)
*p-*Value	0.259	0.004	0.068	0.012
**Stress reduction**			
YES	1.49 (1.03–2.19)	4.95 (4.26–5.66)	3.00 (2.44–3.60)	1.35 (1.16–1.57)
NO	1.50 (1.04–2.37)	5.09 (4.31–5.65)	3.00 (2.44–3.60)	1.43 (1.20–1.64)
*p*-Value	0.607	0.381	0.600	0.025

Data was presented in Median (interquartile range), the differences between two groups were compared by Wilcoxon rank sum test.

BPHS, basic public health services; TG, triglyceride; TC, total cholesterol; LDL-C, low-density lipoprotein cholesterol; HDL-C, high-density lipoprotein cholesterol.

### Treatment patterns and hypertension control rate

In Table 4, among 1,011 hypertensive patients included in BPHS management, 724 (71.6%) took medication, 549 (54.3%) chose monotherapy, and 175 (17.3%) used a combination of antihypertension drugs. All the treatment proportions were higher than the patients without BPHS coverage. In patients without taking antihypertensive medication, the control rate was higher in the BPHS group compared with the non-BPHS group (26.1 vs. 9.8%, *p* < 0.001).

**Table 4:** Number of antihypertensive medications used and hypertension control rate between the BPHS and non-BPHS groups.

**Number of drugs**	**Proportion**			**Control rate of hypertension**		
	**BPHS group**	**non-BPHS group**	***p*-Value**	**BPHS group**	**non-BPHS group**	***p*-Value**
0 (Not taking medicine)	287 (28.4)	276 (54.1)	<0.001	75 (26.1)	27 (9.8)	<0.001
1	549 (54.3)	190 (37.3)	<0.001	189 (34.4)	66 (34.7)	0.853
2	151 (14.9)	41 (8.0)	<0.001	63 (41.7)	19 (39.0)	0.596
≥3	24 (2.4)	3 (0.6)	<0.001	9 (37.5)	0 (0.0)	0.529 ^#^

# Fisher's exact test.

### Blood pressure management status distributions among subjects

**Figure 6:**
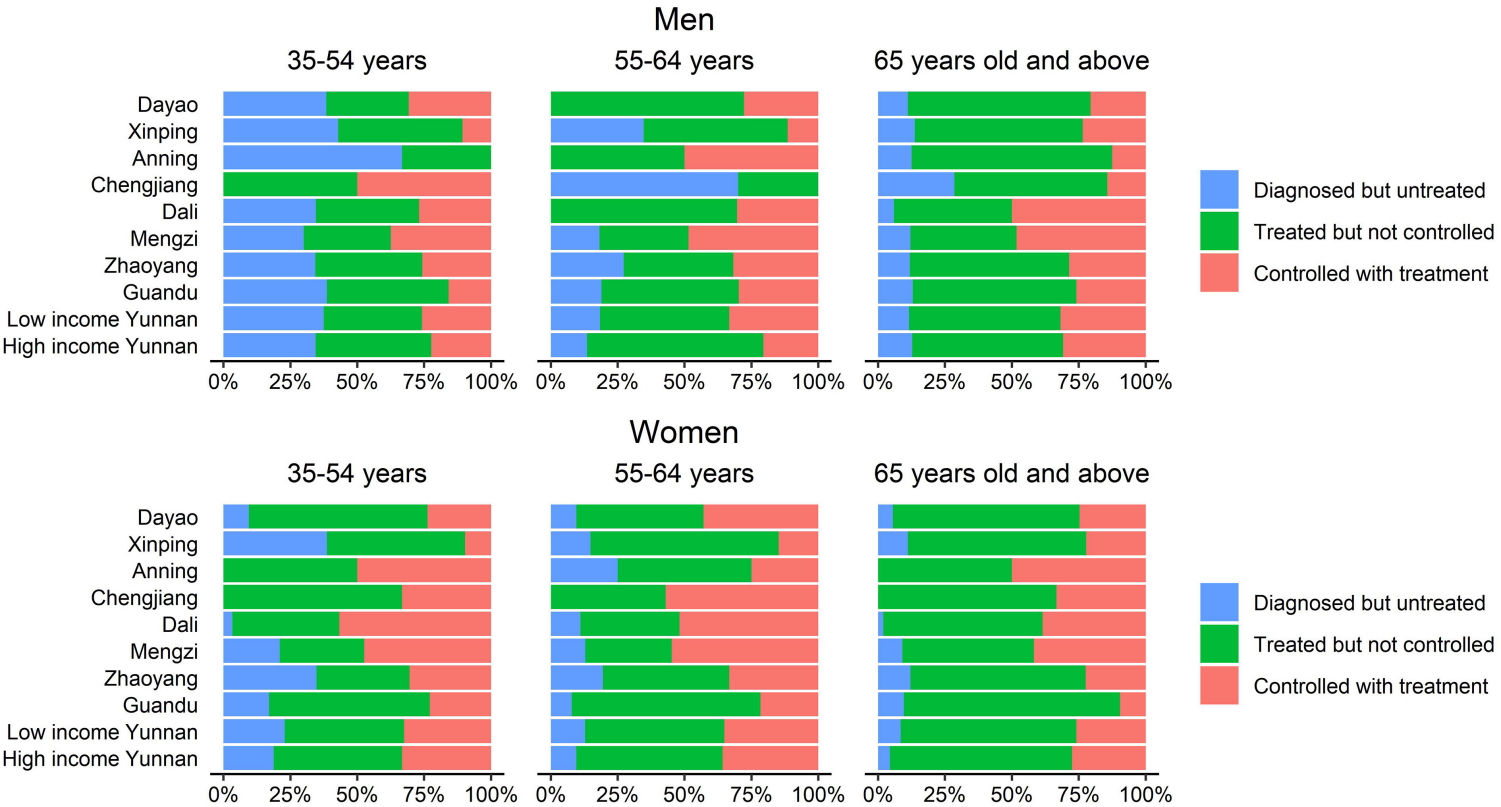
Distribution of blood pressure management status of hypertensive patients in Yunnan.

A lower blood pressure control rate was observed from male patients aged 65 years and above, especially at Dayao, Xinping, Anning, and Chengjiang counties in rural areas. In general, it was observed that, there was a higher prevalence of hypertension treatment and better control among women than men. Patients with hypertension of any gender who are older than 65 can access antihypertensive medications and have better control over their condition. There is no significant difference noticed between subjects with different income levels among the groups of “diagnosed but untreated,” “treated but not controlled,” and “controlled with treatment.” (Figure 6).

## Discussion

This study showed the status of BPHS coverage and the effectiveness in the management of hypertensive patients in Southwest China (Yunnan Province) was corroborated by provincial-specific investigation results. Patients' ID in the survey matched with the BPHS system and found that 66.5% of hypertensive patients were included in BPHS and received related management services. The accessibility of BPHS for hypertensive patients in Yunnan Province was higher than the national accessibility rate (26). BPHS in this province is a patient-centered system which has access to multiple medical databases (including medical institution outpatient and inpatient case databases, electronic medical examination records, etc.) and is thence based on individual care. Due of this well-organized system, more hypertensive patients will be identified proactively compared to other provinces. In the event a patient is identified as hypertensive by the system, a primary care physician will provide continuous BPHS management services. However, the BPHS accessibility rate was found to be lower than that in China's developed regions (90%) (37), as Yunnan province in southwest China has a relatively backward economy, 94% of its terrain is alpine, and 33% of its population is ethnic minority, which could be a plausible reason for relatively low BPHS access. The community uses BPHS or a comparable information system to compile data on daily medical services provided to the community, hypertension management and treatment results are yet to be improved with limited resources. In order to effectively treat all patients with hypertension, particularly in rural and remote regions of low-income provinces in southwest China, there is a need to enhance access to public and primary health services for these patients (12, 38). It is imperative that this regional gap in BPHS be addressed with utmost importance and urgency.

Uncontrolled hypertension may result in ~24,914,000 years of life lost and 28,657,000 quality-adjusted life years lost in 1.7 million Chinese adults (39). Therefore, it is dire and imperative to ensure widespread BPHS accessibility and coverage. In addition, hypertensive patients have multiple risk factors (27, 40), and multiple cardiovascular disease risk factors will proportionately increase the risk of coronary artery, cerebrovascular, and renal disease (27, 29). An important part of BPHS is to assist with interventions of modifiable risk factors for hypertensive patients, including salt reduction, regular physical activity, weight reduction, smoking cessation, alcohol restriction, and reduction of psychological stress, consistent with the global practice guidelines developed by ISH (27), American College of Cardiology (41) and with the support of extensive research evidence done on hypertension (8, 42–46). We also found hypertensive patients in the BPHS group had lower rates of smoking and drinking. Furthermore, as one of the indicators of the level of cardiovascular risk (29), patients included in BPHS management were beneficial in reducing TC after receiving lifestyle modification services. In comparison, the BPHS group had greater percentages of overweight and obese people, smokers, alcoholics, insufficient exercisers, and insufficient consumers of vegetables and fruits. All these are attributable to high lipids. At the same time, challenges remained as 42% of doctors in China's township health centers have only a college degree or less in 2018 (39). The average village clinic in Yunnan Province has two doctors who are required to provide daily consultation services and regular primary health care services for villagers, including the management of 180 hypertensive patients (7). These primary health care providers were faced with a heavy workload and had to compromise on quality in order to meet the quotas set by provincial Basic Public Health Services (18). Even though BPHS improved the service results, it still could not keep up with the fluctuating demand (18). The promotion of lifestyle modifications among high-risk populations under the guidance of BPHS in Yunnan Province has to be further enhanced, undoubtedly.

The study also found, in the BPHS group, 71.6% of hypertensive patients used antihypertensive medication (vs. non-BPHS 45.9%), which was higher than the national treatment rate of 61.3% (12). It may be related to the fact that the BPHS system in Yunnan Province has linked multiple medical electronic databases, benefiting from the management of more patients. Such patients receiving more BPHS accessible health education services, a better awareness of their health, and improved medication adherence, similar to the study in Zhejiang Province and Jiangsu Province in China (25, 47). Moreover, 17.3% of the BPHS patients used a combination of antihypertension drugs (vs. non-BPHS 8.6%), which has facilitated blood pressure control to normal in patients in the BPHS group. However, there is still room for improvement in the BPHS, as evidenced by the fact that in Yunnan Province especially in rural districts, only 39% of antihypertensive drug users had visited a doctor or taken medication in the previous 3 months (48).

Generally, the control rate of hypertension is the standard for evaluating the effectiveness of interventions, resulting in effectively preventing or delaying the occurrence of stroke, myocardial infarction, heart failure, renal insufficiency, and other complications (29). Our study revealed that the BPHS group had a significantly higher control rate, and the multivariate regression analysis found the BPHS group has a 4.8 times more likely chance of having a high blood pressure control rate than the non-BPHS group (OR = 1.640, 95% CI: 1.237–2.175). Moreover, in comparison with hypertensive patients in the non-BPHS group, the hypertension control rate for all ages among those who received BPHS management was significantly higher. Those who participated in BPHS management (26.1%) had a higher control rate than those who did not include in BPHS management (9.8%) despite untreated with hypertension medication, which may be explained by the blood pressure monitoring, risk factor intervention and referral services provided by primary care physicians in BPHS. The results also supported earlier studies indicating that in Yunnan province, women were more aware of and controlled their hypertension than men (48). Besides, hypertensive patients under 65 more unlikely being managed by BPHS in rural areas. It suggests the importance of early screening of younger patients and makes BPHS more capable of providing for the concerns of the younger population to control the morbidity of hypertension at a very early stage. The fact that more than half of our subjects had completed middle school or less and 76.3% of them had household incomes of <USD 4487/year illustrated the validity of the BPHS, the critical role it plays in early screening, prevention and control of hypertension in areas with limited resources, and the ability to share the experience with other developing nations.

Limitations include the design method in this survey, this might not substantiate causation, particularly the link between the services offered by the BPHS program and the management of hypertension without these services. Recall bias can also be a challenge with self-reported data. Some subjects lacked information, such as height or weight, which can skew the data. Therefore, information collection and interpretation of risk factor changes should be conducted with utmost care.

## Conclusions

Since its launch in 2009, the BPHS program run by provincial authority healthcare providers has made a substantial contribution to bettering hypertension management and balancing access to healthcare across inhabitants of urban and rural areas as well as among various socioeconomic strata.

Results showed that program participants kept healthy lifestyles, had lower blood pressure, higher control rate of hypertension, and took their medications more consistently, which could account for some of the benefits of this program. In districts or counties with fully operational digitalized and centralized data management systems, it was easier to include the participants compared to “non-matched ID” in rural areas, therefore health information management may further be improved to address this limitation in rural areas. In order to better diagnose and treat hypertension at its early stages, a customized program has to be designed to cater to the requirements of hypertension control in the younger population through the BPHS program.

## Data availability statement

The raw data supporting the conclusions of this article will be made available by the authors, without undue reservation.

## Ethics statement

The studies involving human participants were reviewed and approved by the central Ethics Committee at the Fuwai Hospital, CAMS/National Center for Cardiovascular Diseases approved this project (approval no. 2020–1360, approval date: August 11, 2020). The survey was conducted in accordance with the ethical principles of the Declaration of Helsinki, and the informed consent and electronic signature of the participants were obtained before the investigation. The patients/participants provided their written informed consent to participate in this study.

## Author contributions

LP and LK were co-first authors and drafted the manuscript. ZG and YZ initiated, conceived, and supervised the study. LD, WL, and MF guided the analysis and modified the article. LP, JF, and HF were involved in data curation and analysis. YS and MT completed field execution and coordinated all divisions. XW, MM, and HW checked the integrity of the data. LK and DM reviewed and edited the manuscript. LD conceptualized and supervised this project. All authors have read and approved the final version of the manuscript.

## Funding

This work was supported by Key Research and Development Program from Yunnan Province Science and Technology Department (Grant No. 202103AF140002); Yunnan Provincial Clinical Research Center for Cardiovascular Diseases-New Technology Research and Development Project for Diagnosis and Treatment of Major Cardiovascular Diseases (Grant No. 202102AA310002); and Provincial Innovation Team Project of Heart Failure Diagnosis and Treatment in Fuwai Yunnan Cardiovascular Hospital (Grant No. 202005AE160020).

## Conflict of interest

The authors declare that the research was conducted in the absence of any commercial or financial relationships that could be construed as a potential conflict of interest.

## Publisher's note

All claims expressed in this article are solely those of the authors and do not necessarily represent those of their affiliated organizations, or those of the publisher, the editors and the reviewers. Any product that may be evaluated in this article, or claim that may be made by its manufacturer, is not guaranteed or endorsed by the publisher.

## Supplementary material

Supplementary file 1 Questionnaires.

Supplementary file 2 Visual abstract - Summary of the investigation process of this study.
