# Supplementary material for: Dawning public health services dogma: An indigenous Southwest Chinese perspective in managing hypertension-with or without the “BPHS”?
Source: Front Public Health. 2022 Nov 10;10:1017795. doi: 10.3389/fpubh.2022.1017795 (PMC9686286; doi:10.3389/fpubh.2022.1017795)
Supplement: Supplementary file 1 — Questionnaires. [file Data_Sheet_1.docx]

Supplementary Material

**This questionnaire was framed by the National Center for Cardiovascular Diseases/** **Fuwai Hospital, CAMS, China**

**Part I Demographic Information**

**A1 Province District / County Street (township/township) residence/village committee**

ID Number:

**A2 Name:**

**A3 Gender:** 1=Male 2=Female

**A4 Date of birth：** MM DD YY

**A5 Ethnicity:**

1 =Han Chinese 2= Mongolian 3= Hui 4=Tibetan 5 =Uighur 6 =Miao 7=Yi

8 =Zhuang 9 =Buyei 10 =Korean 11 =Manchu 12 =Dong 13=Yao

14 =Bai 15= Tujia 16= Hani 17 =Kazakhs 18 =Da 19 =Li

20 =Other ethnic groups (specify )

**A6 Education:**

1=Not attending school 2=Elementary school 3=Junior high school

4=High school/Junior college 5=College/Undergraduate 6=Graduate

**A7 Marital status:**

0=Unmarried 1=Married/Remarried/Cohabiting 2=separated 3=divorced 4=widowed

**A8 Types of medical insurance system (multiple answers allowed):**

1=Medical insurance for urban workers 2 = New agricultural cooperation

3=Medical insurance for urban residents 4=Commercial medical insurance

5=Publicly funded medical care 6=Other

**A9 Employment status:**

1=Working 2=Retired 3=Student (*Jump to A11*)

4=Unemployed or unemployed (*Jump to A11*)

**A10 Occupation**

1=Organization, enterprise and institution managers 2=Professional and technical personnel 3=General clerical and related personnel 4=Commercial, service industry personnel

5=Self-employed industrial and commercial workers 6=Non-farm household industrial personnel

7=Farmers engaged in non-agricultural work 8=Agricultural laborers (engaged in agriculture, forestry, animal husbandry and fishery) 9=Other

**A11 Including yourself, how many members are there in your family?**  **A12 What is the range of disposable/net income per capita of your family in a year last year (RMB)?**

1= <10,000 2=10,000-20,000 3=2-3,000 4=3-5,000 5= 5-10,000 6= ≥100,000

**Part Ⅱ Personal health behavior**

**B1 Smoking (including tobacco, cigarettes)**

**
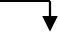
B1.1 Your smoking status in the last 30 days: 0=No smoking (***Jump to* **B1.2)**  1=Smoke every day 2=Smoke, but not every day

| B1.1.1 How long have you been a daily smoker? |
| --- |
| 1=<3 months 2=3-6 months 3=6-12 months 4 = more than 1 year ( years) |

**B1.2 Have you smoked in the past?**

0=No (*(Jump to Drinking alcohol*) 1= Every day 2=Smoke, but not every day

**B2 Drinking alcohol**

**B2.1 Have you ever consumed alcohol in the past?**

0=No (*(Jump to* Diet) 1=Yes

**B2.2 Which of the following categories do your drinking habits fall into?**

1=At least 1 time per day 2=At least 1 time per week 3=At least 1 time per month

4 = Occasional drinking, drinking times per year

5 = Seasonal drinking, drinking months per year, drinking times per month

### B3 Diet (consumption of the following foods in the past month)

| Food | Frequency of consumption | Serving size |
| --- | --- | --- |
| B3.1 Fresh vegetables (excluding dried and pickled vegetables) | 0=Never 1=Day 2=Week 3=Month 4=Year | pounds taels |
| B3.2 Fresh fruit | 0= Never 1=Day 2=Week 3=Month 4=Year | pounds taels |

**B4 Physical Activity**

**B4.1 During the past month, how many days in a typical week did you engage in moderate intensity activity or more?** (Moderate intensity activities are those that require moderate physical effort such as washing clothes, scrubbing windows, cleaning floors, hoeing, fertilizing, playing tai chi, etc., or activities that cause a mild increase in respiration and heart rate such as brisk walking, rice-chanting, dancing, etc.)

Days

**B4.1.1 What is the average cumulative time per day for the above activities?**

Hours minutes

**Part III Information about previous hypertension**

C1 Have you been diagnosed with hypertension?

0=No (*(Jump to* Part Ⅳ Physical and biochemical examinations) 1=Yes


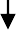


2=Don't know (*(Jump to* Part Ⅳ Physical and biochemical examinations)

| **C1.1 How often did you take antihypertensive medication during the two weeks?**  0=Not taking medicine 1=Occasional (<5 days/week) 2=Regular |
| --- |
| **C1.2 The antihypertensive drug you are currently taking is:**  A  B  C  D |
| **C1.3 Do you have medical staff to provide you with hypertension management services?**  0=No 1=Yes |
| **C1.4 Have you received any of the following instruction (multiple answers allowed)?**  1=Salt restriction 2=Regular exercise 3=Weight control 4=Smoking cessation 5=Alcohol restriction 6=Psychological guidance  **C1.5 In what way do you receive information on the prevention and treatment of hypertension? information (multiple answers allowed)?**  1=TV/Internet education 2=Offline health classes 3=Mobile phone text or WeChat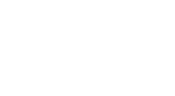 4=Newspaper books 5 = Face-to-face consultation with medical staff 6=Other |

Part Ⅳ Physical and biochemical examinations

**D1. Physical tests**

D1.1 Height: ________cm

D1.2 Body weight: ________kg

D1.3 Waist circumference: ________cm

**D2. Blood pressure**

D2.1 First systolic blood pressure: ________mm Hg

D2.2 First diastolic blood pressure: ________mm Hg

D2.3 Second systolic blood pressure: ________mm Hg

D2.4 Second diastolic blood pressure: ________mm Hg

D2.5 Third systolic blood pressure: ________mm Hg

D2.6 Third diastolic blood pressure: ________mm Hg

**D3. Blood lipids**

D3.1 Total cholesterol: _________mmol/L

D3.2 High-density lipoprotein cholesterol: _________mmol/L

D3.3 Triglycerides: ___________mmol/L

D3.4 LDL cholesterol: _________ mmol/L
