# Supplementary material for: Dawning public health services dogma: An indigenous Southwest Chinese perspective in managing hypertension-with or without the “BPHS”?
Source: Front Public Health. 2022 Nov 10;10:1017795. doi: 10.3389/fpubh.2022.1017795 (PMC9686286; doi:10.3389/fpubh.2022.1017795)
Supplement: Supplementary file 2 — Visual abstract - Summary of the investigation process of this study. [file Data_Sheet_2.pdf]

Dawning Public Health Services Dogma: an Indigenous South-West Chinese Perspective in Managing Hypertension - with or without the 'BPHS'?

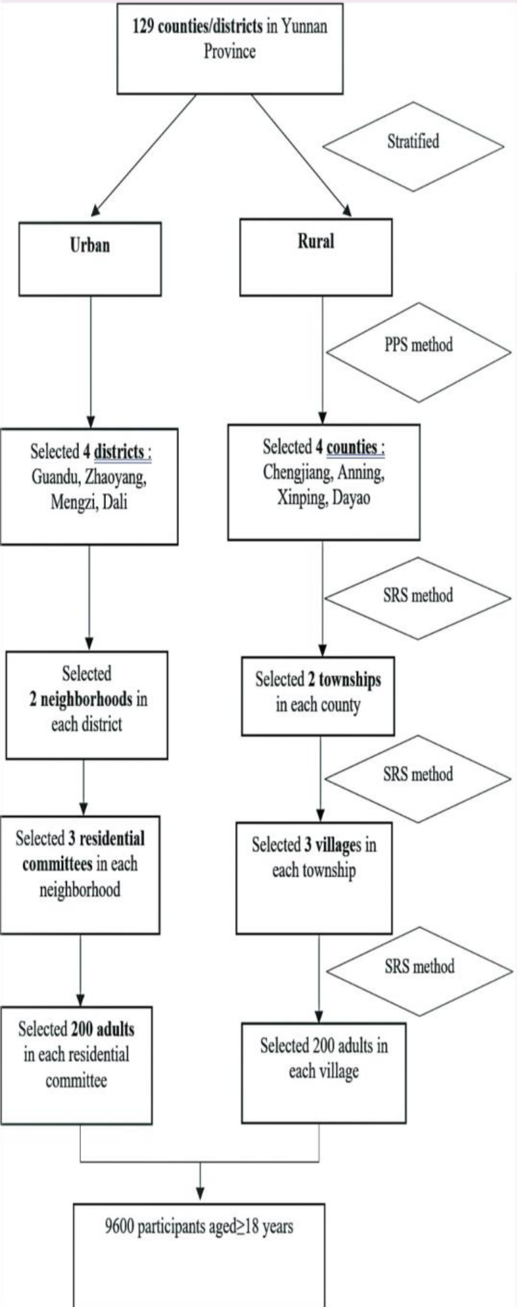

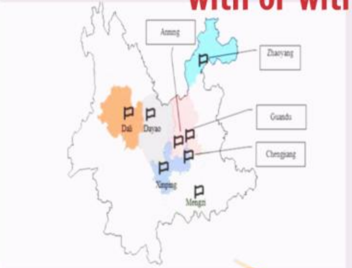

6456 Participants
selected from 9600 in 8 regions of Yunnan

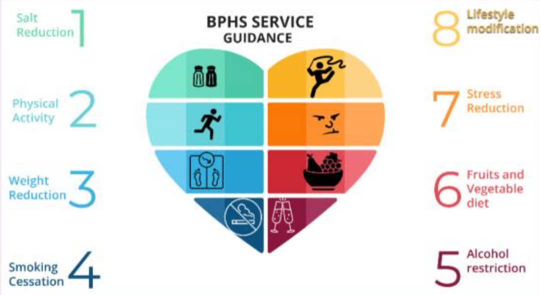

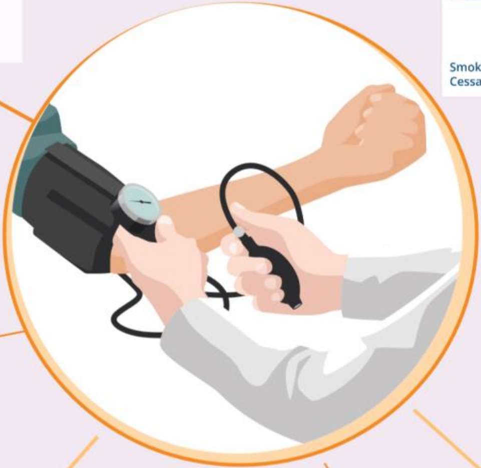

Informed consent

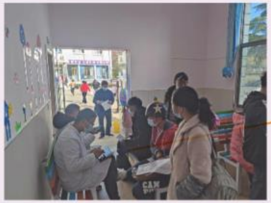

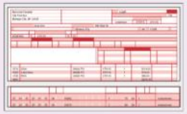

Results

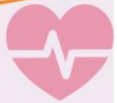

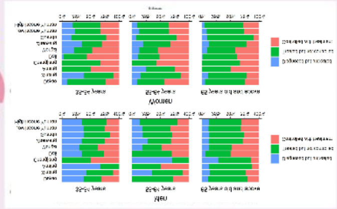

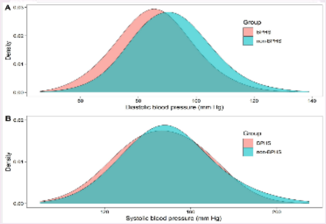

Weight Check

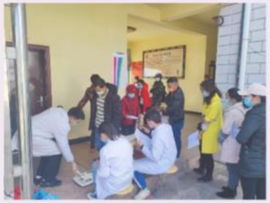

BMI check

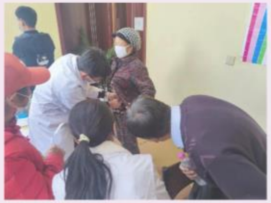

BP check

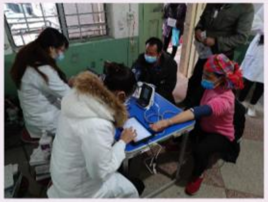

Blood sample collection

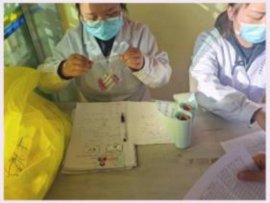

Clinical and Epidemiology Project lead: Duo Lin
All Correspondence to : duolin@hotmail.com
Affiliated Cardiovascular Hospital of Kunming Medical University, Fuwai Yunnan Cardiovascular Hospital, Kunming 650221, China
